# Facilitators and Barriers to the Implementation of BETTER WISE, a Chronic Disease and Prevention Intervention in Canada: A Qualitative Study

**DOI:** 10.1007/s43477-023-00074-7

**Published:** 2023-03-16

**Authors:** Nicolette Sopcak, Carolina Fernandes, Daniel Ofosu, Melanie Wong, Ielaf Khalil, Tracy Wong, Donna Patricia Manca

**Affiliations:** 1 Department of Family Medicine, University of Alberta, 6-10 University Terrace, Edmonton, AB T6G 2T4 Canada; 2 Lunenfeld-Tanenbaum Research Institute, Sinai Health System, Toronto, ON Canada; 3 Strategic Clinic Networks, Alberta Health Services, Calgary, AB Canada; 4 Covenant Health, Grey Nuns Community Hospital, 1100 Youville Drive Northwest, Edmonton, AB T6L 5X8 Canada

**Keywords:** Implementation, Primary prevention, Chronic disease, Primary care, Patient care team, Qualitative research

## Abstract

The aim of the BETTER WISE intervention is to address cancer and chronic disease prevention and screening (CCDPS) and lifestyle risks in patients aged 40–65. The purpose of this qualitative study is to better understand facilitators and barriers to the implementation of the intervention. Patients were invited for a 1-h visit with a prevention practitioner (PP), a member of a primary care team, with specific skills in prevention, screening, and cancer survivorship. We collected and analyzed data from 48 key informant interviews and 17 focus groups conducted with 132 primary care providers and from 585 patient feedback forms. We analyzed all qualitative data using a constant comparative method informed by grounded theory and then employed the Consolidated Framework for Implementation Research (CFIR) in a second round of coding. The following key elements were identified: (1) Intervention characteristics—relative advantage and adaptability; (2) Outer setting—PPs compensating for increased patient needs and decreased resources; (3) Characteristics of individuals—PPs (patients and physicians described PPs as compassionate, knowledgeable, and helpful); (4) Inner setting—network and communication (collaboration and support in teams or lack thereof); and (5) Process—executing the implementation (pandemic-related issues hindered execution, but PPs adapted to challenges). This study identified key elements that facilitated or hindered the implementation of BETTER WISE. Despite the interruption caused by the COVID-19 pandemic, the BETTER WISE intervention continued, driven by the PPs and their strong relationships with their patients, other primary care providers, and the BETTER WISE team.

## Contributions to the Literature

- Despite the constant rise of chronic diseases, research suggests that it is difficult and time consuming for primary care providers to address prevention and screening in primary care
- This qualitative study contributes by offering an in-depth and comprehensive understanding (including perspectives from patients, physicians, and other health professionals) of how a prevention and screening intervention can be implemented despite the challenges of an ongoing global pandemic.
- Prevention practitioners, specifically trained members of a healthcare team, were able to harness relationships between different stakeholders (patients, physicians, other health professionals) to drive implementation of the intervention as “agents” of implementation.

## Background

Chronic diseases like cancer, cardiovascular disease, and diabetes kill about 28.7 million people annually and are an increasing problem worldwide (World Health Organization, 2022). Although early detection, screening, and a healthy lifestyle are known components to curb this risk, people are often unaware of how to decrease their personal risk and what steps they can take in that direction. Primary care providers lack the time to address chronic disease prevention and screening (Shein & Stone, 2017). The BETTER WISE (Building on Existing Tools to Improve Cancer and Chronic Disease Prevention and Screening in Primary Care for Wellness of Cancer Survivors and Patients) intervention is a comprehensive and structured approach that proactively addresses cancer and chronic disease prevention and screening (CCDPS), including cancer survivorship and screening for poverty and lifestyle risks for patients 40–65 years of age (Manca et al., 2018). BETTER WISE is a Canadian-based cluster randomized controlled trial (cRCT) that includes a qualitative evaluation and economic assessment (Manca et al., 2018). BETTER WISE evolved from several previous BETTER studies, which have shown improved chronic disease prevention and screening outcomes and patients’ appreciation of this approach (Aubrey-Bassler et al., 2019; Grunfeld et al., 2013; Lofters et al., 2021; Manca et al., 2014; Sopcak et al., 2016, 2017).

For the implementation of BETTER WISE, 1005 patients from 13 primary care clinics (urban, rural, and remote) in three provinces in Canada (Alberta [AB], Ontario [ON], and Newfoundland and Labrador [NL]) were invited for a one-hour prevention visit with a prevention practitioner (PP). PPs were allied health professionals (e.g., nurses, dietitians) who were part of a primary care team who received training in cancer and chronic disease prevention and screening, cancer survivorship, and the BETTER WISE approach including tools and resources (Manca et al., 2014, 2015). PPs were selected based on availability and capacity to take on the role of PP in addition to their regular workload. The one-hour prevention visit was a one-on-one, personalized visit which focused on eligible screening, lifestyle counseling, and provided patients with the opportunity to set goals. In addition, survivors of breast, colorectal, and/or prostate cancer also received individualized counseling on cancer surveillance (Manca et al., 2018). Patients aged 40 to 65 were randomized into receiving either a prevention visit or standard care (i.e., wait-list control). The primary outcome was measured approximately 12-months after patients received their prevention visit using a composite index (Manca et al., 2018). This qualitative study describes the implementation of the BETTER WISE intervention, including the facilitators and barriers as perceived by primary care providers, clinic staff, allied health professionals, and patients.

## Methods

### Setting and Participants

Thirteen primary care clinics across Canada (Alberta (AB), Ontario (ON), and Newfoundland and Labrador (NL)) participated in the BETTER WISE cRCT. Each clinic selected their own prevention practitioner (PP), an allied healthcare provider, which in total included three registered nurses, four licensed or registered practical nurses, one nurse practitioner, one registered dietitian, one pharmacist, one clinical medical assistant, one kinesiologist, and one clinic coordinator. Before implementation of the program, these individuals received training from the BETTER WISE team on the PP role and current clinical guidelines and recommendations for primary prevention and screening of cancer and chronic disease as well as cancer survivorship for patients with a personal history of breast, colorectal, and/or prostate cancer. Additionally, PPs were also trained on the BETTER WISE toolkit and certified in Brief Action Planning, a structured approach informed by the spirit of motivational interviewing to guide patients to make S.M.A.R.T. (Specific, Measurable, Achievable, Relevant, and Time-Bound) health goals (Centre for Collaboration, Motivation, & Innovation, 2022).

One-thousand-five patients agreed to participate in the BETTER WISE study. Patients were randomized to either receive a prevention visit with the PP at time of enrollment (the intervention group) or to a wait-list control group, who received their prevention visit approximately 12-months after their enrollment in the study. Patients in the intervention group also received follow-up visits with the PP every 6 months for up to 2 years and all visits were expected to take place primarily in-person.

Prior to a prevention visit, PPs reviewed patients’ medical charts and answers to the BETTER WISE survey, which included questions on health status, family and personal medical history, social determinants of health (e.g., screen for poverty and food security), and lifestyle behaviors (smoking, alcohol, physical activity, and diet). Together, PPs and patients discussed the patients’ health status, what screening they were eligible to receive, and whether they wanted to set a specific health plan (e.g., lifestyle modifications such as reducing or quitting smoking, eating more vegetables, and increasing physical activity levels). The PPs also referred patients to appropriate resources (e.g., cancer screening program, smoking cessation program, social worker, counselor) and back to patients’ primary care providers for further assessment or treatment, if needed (Manca et al., 2018). Details of the quantitative results of the trial will be published elsewhere (Manca et al., 2023).

For the qualitative study, we used convenience sampling and sent out emails to all 13 participating primary care sites, inviting PPs, primary care providers and their staff to focus groups and key informant interviews at three different time points of the study: at the beginning (mid to late 2018), mid-way through the study (mid to late 2020), and at the end of the implementation (early to mid-2021). All baseline focus groups took place in person at the respective clinic site and took about 60 min on average. After the onset of the coronavirus disease (COVID-19) pandemic in March 2020, focus groups took place online over Zoom® and all key informant interviews were conducted either over the telephone or virtually (Zoom®), based on each participant’s preference. Follow-up focus groups and interviews were a bit shorter, on average between 40 and 50 min. Written consent was obtained from all participants either in-person or via e-mail. We used a semi-structured interview guide to learn more about each primary care setting’s context, process, and how the implementation (including facilitators and barriers) of BETTER WISE impacted patients and providers. Patients were invited to provide written feedback after the visit if they wished to do so. The feedback was collected anonymously (the feedback form did not include their name or any identifiable information), and patients could leave their feedback in a box located in the clinic waiting room or send the feedback to the research team in a pre-stamped, pre-addressed envelope. Along with the feedback form, patients also received an information letter, which informed them that by completing and submitting the feedback form, they were providing implied consent to participate in the qualitative component of the study.

Ethical and operational approvals were granted by the Health Research Ethics Board at the University of Alberta (Pro00067811 and Pro00069064), the Health Research Ethics Board of Newfoundland & Labrador (#2017.027, 2017.027B, 2017.027C, 2017.027D, and 2017.284), the Markham Stouffville Hospital Research Ethics Board (no file number assigned), and the Research Ethics Board at St. Michael’s Hospital (#17-050 and #17-248). All methods were performed in accordance with the relevant guidelines and regulations. Focus groups and key informant interviews were conducted and audio recorded by the first author, NS, a PhD-level trained researcher and qualitative research lead for BETTER WISE. For verbatim transcription, we used the transcription software Trint® and both NS and DO (research assistant for BETTER WISE) proofread and edited all transcripts. We also collected field notes and memos (descriptive and conceptual records of data) for contextualization. Patient feedback forms were entered by DO (research assistant) and MW (study coordinator) into REDCap®, an electronic data capture tool hosted and supported by the Women and Children’s Health Research Institute at the University of Alberta, and exported to a generated Excel spreadsheet (Microsoft® Excel, Version 16.20).

### Data Analysis

We conducted a two-stage qualitative analysis. In the first stage, NS took the lead in the first round of coding to analyze all qualitative data using the constant comparative method informed by grounded theory (Glaser & Strauss, 1967). Codes were created, that is, a word or phrase was assigned to each line or paragraph, which were then used to conceptualize the data into a larger theme. When new data came in, new codes were created and compared to the existing codes and themes in an iterative process. (Glaser, 1998). A qualitative methods team consisting of both male and female members with diverse professional backgrounds and experiences (NS, DM, CF, MW, DO, IK, TW) met several times to review and discuss the data, the coding process, and revise emerging themes. Team members looked at the data independently before the team meetings and each theme was revised until we found consensus. The team used reflexivity by remaining open to different interpretations and verifying that conceptualizations of themes were sufficiently supported. In the second stage, we employed the original version of the Consolidated Framework for Implementation Research (CFIR) in a second round of coding as an overarching typology to focus our analysis on the most salient categories of the five CFIR domains and identify the barriers and facilitators of the implementation of BETTER WISE: (1) Intervention characteristics, (2) Outer setting, (3) Characteristics of individuals, (4) Inner setting, and (5) Process (Damschroder et al., 2009). We chose this framework as the five CFIR domains are accessible and concise, have been consolidated by a synthesis of existing frameworks, and are recognized as the main elements in program implementation in the health sciences (Damschroder et al., 2009; Kadu & Stolee, 2015). In qualitative research, data saturation occurs when no new or relevant data emerges. We had a large number of data points across many data collection periods, with 17 focus groups and 48 key informant interviews (*n* = 132 participants) and 585 patient feedback forms returned. We also focused our analysis on the most salient concepts where data saturation was reached.

### Rigor

To ensure rigor, we collected data from 13 different primary care clinics in three different Canadian provinces (including urban, suburban, rural, and remote settings) and at three different data points over the span of three years during implementation. We also captured a variety of perspectives by including primary care providers, clinic staff, allied health professionals, and all PPs as well as feedback from patients, so that we could triangulate and compare these different perspectives. Using a variety of data collection strategies (focus groups, key informant interviews, and feedback forms) also allowed us to accommodate participants’ schedules and preferences. Lastly, our research team was involved in analysis meetings to discuss the data and the emerging themes until consensus was reached.

## Results

A total of 132 primary care providers and their staff (i.e., physicians, PPs, other health professionals) from 13 primary care settings participated in 17 focus groups (*n* = 128, 16% male (*n* = 20), 84% female (*n* = 108); 54% from AB (*n* = 69), 30% from ON (*n* = 38), and 16% from NL (*n* = 21)) and 48 key informant interviews (*n* = 25, 2 men, 23 women; 12, 7, and 6 from AB, ON, and NL, respectively). Since data collection took place at three different points in time and participation was open to everyone involved in the implementation of BETTER WISE, some participants were involved in both interviews and focus groups. Patient perspectives were included through the 585 written feedback forms collected from patients who were invited for a prevention visit with a PP. Thirty-six percent of patients who provided feedback were male (*n* = 210) and 62% (*n* = 363) were patients at clinics in Alberta, Canada (19% were from ON (*n* = 111) and 19% were from NL (*n* = 111)). In line with the CFIR, we present our results using the following five domains: (1) Intervention characteristics, (2) Outer setting, (3) Characteristics of individuals, (4) Inner setting, and (5) Process. The five domains with domain subcategories and respective key facilitators and barriers to the implementation of the BETTER WISE intervention are presented in Table 1 and described in greater detail below.

**Table 1:** CFIR domains of BETTER WISE implementation

CFIR domain	Key Element	Barrier [potential impact]	Facilitator [potential impact]
Intervention characteristic	Relative advantage	• Lack of buy-in from physicians [protective of their process, late or non-adopter of intervention]	• Primary care providers’ perception that intervention is beneficial to patients and prevents patients from “falling through the cracks” [perception of improved prevention and/or early detection of chronic conditions and cancers] • Intervention is comprehensive and allows enough time for PPs to help patients with screening timelines, health goals, and resources [patients’ questions addressed improving knowledge of health and resources]
	Adaptability (implementation of intervention was underway when COVID-19 pandemic was declared)	• Some patients could not be reached by phone • Some patients and PPs preferred in-person [unable to access some patients and difficult to build rapport]	• PPs’ ability to adapt BETTER WISE visits • Patients appreciated receiving visits over the phone, felt supported [perceived to be efficient, convenient and allowed for timely check-ins]
Outer setting	Patients’ needs and resources	• Staff shortages, lack of resources at clinic setting • Patients had only limited access to preventative care, screening tests were delayed [delay in prevention and screening]	• Patients’ perception of visits as valuable and helpful • PPs supported patients with goal setting (some goals changed due to COVID-19 related issues)[perceived to help inspire health lifestyle choices and reduce isolation]
Characteristics of individuals^a^	Prevention practitioners (PPs)	• Not finding a replacement when PP left role [loss of skilled role]	• PPs enjoyed their role and found it rewarding • Physicians valued PPs and supported them • Patients appreciated PPs’ personal attributes (e.g., knowledgeable, helpful, compassionate) and how visit made them feel (e.g., listened to, not rushed) [access to skilled individuals with excellent interpersonal skills committed to chronic disease prevention and screening]
Inner setting	Network and communication	• Lack of support with admin tasks [limits ability to spend time with patients] • PP outsider to clinic / lack of connection patients [perceived difficulty to develop personal connections with patients]	• PPs perceived to be supported by physicians and managers, collaborative team effort, team leaders navigating pandemic [improved communication and support to each other]
Process	Executing	• Pandemic-related issues hindered execution [postponed or missed opportunities for prevention and screening]	• Some PPs had increased availability to continue with visits • BETTER WISE team support to PPs, adapting to changes [improved exchange of information on how to navigate the situation]

^a^Although we tried to fit the “Characteristics of Individuals” in one of the CFIR constructs, we found that neither of them reflected the key individual characteristics. Assigning our PPs their own domain seemed to be the best and truthful fit.

### Domain 1: Intervention Characteristics

Based on the emerging data, both *relative advantage* and *adaptability* were the two predominant intervention characteristics of BETTER WISE.

#### Relative Advantage

Relative advantage refers to stakeholders’ perception of the advantage of implementing the intervention as opposed to an alternative or existing program. One of the barriers that emerged was a lack of buy-in of primary care providers who were unsure of the advantage of implementing BETTER WISE or did not view it in a positive light. One of the PPs described feeling like some physicians were protective of their process and their patients and that “whatever they’re doing for their follow-up or for their screening is what needs to be done.”

However, a key facilitator was that the majority of respondents considered having BETTER WISE at their clinic an advantage because it prevented patients from “falling through the cracks” and extended the time frame that patients could spend with a healthcare professional (up to 1 h). Respondents also commented on the advantage of holistic and comprehensive patient care, such as helping patients identify screening they were due for, answering health questions, and providing resources for patients. One PP stated that: “[BETTER WISE] definitely prevented a lot of people from falling through the cracks. Because we can still call them to remind them, we are here and try to keep them as up to date as possible.” A physician commented on the PP role at their clinic as follows:

I think having a skilled Prevention Practitioner like [PP] was really terrific. I feel like patients who interacted with [PP] probably had more time to talk through some of their health questions about prevention, and I think [PP] did a much more detailed job, particularly around talking about some of the prevention interventions that would be helpful. And the feedback that I had from patients was that they were really happy with their visit and felt like things were discussed that, I hadn’t done in that same way, and in ways that I think really benefited them (…) Patients were receiving better care than had we not had the BETTER WISE program. [Physician, FG 007, ON]

#### Adaptability

The implementation of BETTER WISE had to be adapted to the new circumstances when the COVID-19 pandemic was declared by the World Health Organization (WHO) in March 2020. The intervention was at a midpoint when prevention visits were shifted from in-person to telephone visits (or virtual visits if clinic protocol permitted). While some patients had been seen by a PP for a prevention visit, other patients had had no interaction with a PP yet, which resulted in PPs having to make “cold” phone calls to new patients who had agreed to participate in BETTER WISE. One of the barriers that primary care providers identified was that it seemed more difficult to reach patients over the phone, as this PP explained: “It was a challenge to get people to answer the phone, answer their surveys, do their visits by phone, things like that and it felt like you were continuously having to call people all the time.”

Some of the patients and the PPs expressed that they preferred in-person visits. One patient reflected on their phone visit and “found it difficult…to do visits over the phone [though it was] (totally unpreventable).” PPs commented that in-person visits helped to build rapport, which could be harder to build over the phone, as this PP explained:

I’m not a huge fan of the phone call visits. I just don’t feel as though you get as connected; like I don’t know who these people are. (…) I like to see people and to have the time and I think people relax a little bit more when they can see someone face-to-face, versus over the phone. (…) I just don’t feel that I’m as helpful. [PP, KI026, AB]

Although some patients and PPs expressed that they did not appreciate the shift from in-person visits to phone calls only, calling patients allowed PPs to have timely check-ins with patients. Both, primary care providers and patients shared that they were grateful the intervention could continue and for some patients it was an efficient alternative. A physician shared: “I thought it was great that the interactions with the patients were continued virtually and I think that was good and it minimized the barriers.” Patients also expressed approval for the phone visits as summarized in this quote by a patient: “Great that this can continue—even through this pandemic—and be able to do over the phone.”

Interestingly, the option to have phone visits made it easier and more efficient for some patients to access primary care; some respondents shared appreciating not having to take time off work, not having to drive to the clinic or pay for parking, and that “patients appreciated it because it was more convenient for them.”

### Domain 2: Outer Setting

#### Patients’ Needs and Resources

Patients’ needs and accessible resources changed with the onset of the COVID-19 pandemic. Changes included closures of labs and extended waitlists where screening and labs were no longer open to patients. Primary care clinics and providers involved in the implementation of BETTER WISE had additional responsibilities (e.g., screening for and vaccination against the COVID-19 virus) and faced the additional challenge of staff shortages. A PP shared their observation that there were “a lot of contract nurses” in their setting who had never seen the patients at the clinic before and that many of the nurses they knew wanted “to be doing something different and [were] looking for different opportunities.”

Even though PPs tried to compensate for shifting clinic priorities, patients could not come into the clinic unless it was urgent. Many patients did not get their screening tests done in time, not only because labs and screening centers were closed, but also because patients did not feel comfortable coming in or decided to postpone screening tests. A PP reflected that: “Even if it wasn’t formally by the lab thing, ‘we’re not doing prevention screening tests right now’, it was the patients going ‘when things settle down I’m going to get this taken care of’.” Another PP elaborated:

It meant no colonoscopy, there were no mammograms happening for sure, there were no PAP tests being conducted because patients were not allowed to come in for physicals unless there was some kind of medical reason that was urgent that they needed to. But normal routine screening like those three specifically I can think of, were stopped. [PP, KI034, ON]

Due to restrictions surrounding the COVID-19 pandemic, patients encountered wait times or closed labs for screening tests such as mammograms, PAPs, and lab work (Sopcak et al., 2023). This proved to be a main barrier for the implementation of BETTER WISE as primary care providers shifted their focus to acute care issues, vaccination, and COVID-19 testing, resulting in screening and prevention being put on the backburner. One of the physician explained it this way:

I think sort of prevention kind of stuff we did as physicians really took a back seat to a lot of the discussions and of course at the beginning, labs wouldn’t even allow us to do present patients’ stool for blood and mammography, they were just turned away. That was troubling, actually. [Physician, KI035, AB]

Some primary care providers shared their strategies to prioritize patients who were deemed at higher risk and had a higher need to get their screening tests done at specific intervals. One of the physicians explained their approach:

I was prioritizing very deliberately my patients with chronic diseases. And as it related to screening and prevention, prioritizing people who were on our high-risk registry, so people who needed to have PAPs or colonoscopies or mammograms done at specific intervals. We were very attentive to making sure that there were absolutely no breaks in that throughout the pandemic. But in terms of the opportunistic screening and the people coming in for those types of maneuvers, those opportunities were dramatically reduced as a result of the pandemic. [Physician, KI047, AB]

PPs attempted to accommodate patients involved in BETTER WISE, even if it meant working overtime and scheduling their prevention visits on evenings and weekends. PPs’ personal investment in the project was an important facilitator. For instance, at least three of the PPs were contracted to other clinics and sacrificed weekends and evenings to stay involved in BETTER WISE and complete the project.

Although the pandemic shifted the focus toward COVID-19-related issues, patients perceived the prevention visits to be valuable and helpful during the pandemic. Patients expressed that they were struggling with reaching their goals during the pandemic, but that they also appreciated the support they received from the PPs over the phone. One patient shared:

My capacity to follow the eating and lifestyle commitments has waxed and waned through my time in this program, partly due to the effects of the pandemic, but it has been a valuable and steady support having the PP’s check-ins, which give me a lift and inspiration to do my best with this. [Patient, female, AB]

PPs also commented that patients seemed to think about their health, including their mental health, and that phone visits were appreciated during a time of social isolation. One of the PPs reflected on patients’ goals: “It impacted the type of goals people set. These [goals] were more focused on mental health goals, because of the isolation and how to deal with that. The goal setting changed to more Covid–related issues.” Another PP reflected on the importance of connecting with patients during the pandemic, who seemed to appreciate a phone call and felt taken care of, especially older patients: “many of [the seniors] have been isolating and were just lonely. So, that was actually an unexpected positive, which was that opportunity to interact with people over the phone.”

### Domain 3: Characteristics of Individuals

#### Prevention Practitioners (PPs)

PPs were the healthcare professionals who met with patients for the BETTER WISE prevention visit and as such were crucial to the implementation of the intervention. Without the PPs’ commitment, the implementation of BETTER WISE was not possible. One main barrier to implementing the intervention was when a PP left and the clinic site could not find a replacement. One of the physicians saw PPs leaving and having to retrain someone for the role (during the pandemic) as a barrier that compromised the study, “because of this lack of continuity, there was a challenge there in terms of the actual objectives.”

The PPs who remained in their role during the pandemic shared that they appreciated the PP role and their involvement in BETTER WISE. Despite challenges related to the pandemic, PPs made a strong effort to continue with the role, which was an important facilitator to continuing with BETTER WISE. The PPs’ buy-in and belief that the intervention was useful and their enjoyment of the role were main facilitators to ensuring that implementation could continue. One of the PP shared at the end of the project:

I was happy to do it, I really enjoyed it. I got to say these last couple of years doing that, that’s one of the best things that I’ve done (…) I was able to help people. And hopefully make a difference so that they would make some positive lifestyle changes, you know? (PP, KI039, NL)

Physicians also shared that they valued the PPs and were comfortable with their patients having prevention visits with the PP, which was another facilitator of keeping the PPs engaged in BETTER WISE despite the pandemic and other demands. Physicians shared that they saw a benefit in having a PP and in patients having the time to sit down to talk about their health in more depth, as this physician explained:

People really appreciated our Prevention Practitioner. She has a very nice way about her. She’s very fun and person-centered and nice to talk to. And so [patients] appreciated her. They also really appreciated the time. You know it’s quite a long visit, so it’s more time to sit down and talk about these things deliberately than they would usually get just with me. [Physician, KI047, AB]

Patients left glowing reviews for their PPs, emphasizing personal attributes (e.g., “thorough,” “helpful”) and how their visit with the PP made them feel (e.g., “listened to”). One patient commented that they “liked time to review [their] “schedule” of future tests.” Another one stated that their PP was “friendly, professional, compassionate, knowledgeable, informative, addressed concerns, provides solutions. I felt comfortable and never felt like I was being rushed.” Time to discuss medical terms and results was also appreciated as this patient noted: “The Prevention Practitioner was very thorough and took the time to explain everything in detail. She made sure I understood everything.”

### Domain 4: Inner Setting

#### Network and Communication

The inner setting refers to the characteristics of the organization where the implementation takes place. Network and communication refer to the formal and informal roles, relationships and hierarchies, as well as communication preferences within each setting, which are important factors in how physicians work together with PPs and other healthcare professionals. As identified in the previous domain, leadership from physicians and PPs were key facilitators to the implementation of BETTER WISE. One important barrier that the PPs identified in the inner setting was the perceived lack of support with administrative tasks involved in the intervention such as calling patients and sending out invitation letters, which proved to be very time consuming. One PP shared that the administrative duties associated with the study was the most challenging part: “Calling patients, sending letters and things like that, but understanding that it’s a really important part of the study (…) what could have helped me at our site would have been to have more people involved.”

Another barrier that emerged was when a PP was not part of an existing team and had no previous relationships with patients of the primary care clinic, especially when PPs worked from home during the visit and were no longer physically present at the primary care setting and had no personal connections with patients. For instance, one of the PPs never visited the clinic in person and did all of their patient visits (including consent and chart abstractions) virtually over the phone. That PP shared: “I felt like I might not have had so much of a personal connection with patients as if it had been done in person.”

However, the barriers named above were exceptions, since overall, respondents reported a perceived sense of support from, and good communication and collaboration with other clinic members who were able to facilitate the implementation. A PP who witnessed it working well at their clinic noted that it was “a real collaborative effort between everyone at the clinic and it works beautifully.” Good relationships between PPs and physicians was also reinforced by responsiveness to requests as this PP shared: “We know that if we send [the physicians] a message that they’ll get back to us in a timely manner and things like that.”

Another facilitator of improved communication and the clinic network was how clinic teams navigated the challenges of the COVID-19 pandemic. For instance, some clinics started to have specific meetings to provide updates to the team to keep team members in the loop, avoid burnout, and ensure that team members were taking care of themselves. Some primary care teams held specific COVID meetings once a week to debrief and plan for next steps. One of the physician shared how their team focused on wellness:

I think it was something that our unit was cognizant of—that there is concern about burnout and fatigue through the pandemic, without a doubt. How we’ve managed it is, part of our objective for the new year is to really focus on wellness—to the point that we’ve developed a wellness committee, that it is at the forefront, recognizing that we have to make sure that all our providers are taking care of themselves to be able to continue their roles at their full capacity. [Physician, FG006, ON]

### Domain 5: Process

Based on CFIR, process broadly entails the components: planning, engaging, executing, reflecting and evaluating. The concepts planning and engaging were less salient for this intervention since primary care clinics were already on board for the implementation of BETTER WISE. Executing emerged as a key element of the process, particularly because execution was disrupted by adaptations to the implementation that became necessary with the onset of the COVID-19 pandemic. As described under Domain 2: Outer setting, clinic teams shifted their focus to respond to the COVID-19 pandemic, including prioritizing acute care issues, COVID-19 testing and vaccination. Furthermore, clinics had to implement new protocols (e.g., cleaning of practice spaces, triaging patients) and follow provincial guidelines (e.g., postponing prevention and screening).

Due to the extent of the changes related to a worldwide health crisis, the BETTER WISE team expected that the implementation would have to be halted or canceled. However, despite some barriers described earlier (e.g., staff shortage), the BETTER WISE implementation was able to continue. PP respondents shared that for some of them availability increased so that they could continue with prevention visits over the phone.

We didn’t have people coming in so I was actually able to focus a little bit better on the BETTER WISE because, obviously, appointments were a little bit different. So, even though we were short staffed I was able to actually take the time to go work and away in the office and not be interrupted, so that was good. [PP, KI045, AB]

PPs were creative in facilitating prevention visits over the phone by sending patients documents so they would have visuals to refer to and a record of what was discussed during the visit, including next steps in written form. Another facilitator was the BETTER WISE team’s efforts to provide support to PPs and brainstorm solutions when challenges arose. The BETTER WISE team offered regular monthly meetings with PPs, where updates were exchanged and the PPs could ask questions. PPs were also encouraged to reach out to their provincial coordinator when they needed help with navigating occurring issues, which they did, as this PP expressed when reflecting on their experience: “Just having [the BETTER WISE Team], helping us through the process when certain changes do happen such as the pandemic and adapting to that, you guys adapted really well and helped us adapt really well.”

## Discussion

In North America, preventive care is underutilized and only a low percentage of patients receive prevention services despite chronic disease being a burden on patients, their families, and healthcare systems (Levine et al., 2019). Rubio-Valera et al (2014) found in their synthesis of 35 studies that “primary care professionals show resistance” to implementing primary prevention and health promotion activities into practice due to a variety of factors including, but not limited to, healthcare professionals’ beliefs, experiences, and skills in primary prevention and health promotion, lack of time and resources, and the prioritization of disease treatment over prevention. Many of these themes are reflected in our findings. Implementing a prevention and screening program during a global pandemic posed a unique challenge in which barriers and facilitators likely were amplified due to primary care providers’ need to respond to competing healthcare demands. Given these circumstances, it may seem somewhat surprising that the implementation of BETTER WISE was successful and PPs continued to conduct their prevention visits with patients.

What was it that allowed this implementation to succeed? The results of this study suggest that all five domains of the CFIR framework were of importance: (1) Characteristic of the intervention: the perceived relative advantage by providers of BETTER WISE and the adaptability of the intervention (PPs switching from in-person to phone or virtual visits); (2) Outer setting: health providers responding to patients’ needs and the resources available to them; (3) Characteristics of individuals: the PPs were the “agents” driving this implementation; (4) Inner setting: strong network and communication in clinic teams and the perceived collaboration with and support by team members and leadership; and (5) Process: executing this implementation, which included adapting to changing circumstances. If we apply our results framed by the five CFIR domains to a higher conceptual level, the overarching theme that connects all five domains is the concept of *relationships,* with the PPs as “agents of implementation” at its core. The PPs are the change agents, as it was their effort and dedication to continue with prevention visits over the phone or virtually (accommodating patients by sending documents or resources by mail) and connecting with patients during a difficult time when access to primary care settings was limited that drove the study to successful completion. In turn, physicians, who found themselves pulled away from prevention and screening due to the additional demands of COVID-19, were appreciative of their PPs checking in on patients and focusing on preventative care as well as monitoring higher-risk patients. Patient respondents emphasized the importance of the relationship with their PP; commented on PPs’ kindness, professionalism, listening skills; and appreciated the trusting space that these visits provided, finding the information and resources provided to be helpful. The concept of trust has been found to be a critical element in patient and healthcare provider relationships and has been linked to improved health outcomes, uptake of prevention, management of disease, and satisfaction with care provided (Murray & McCrone, 2014). PPs, in turn, commented on feeling supported by their own teams (especially physicians and managers) and valuing the support provided by the BETTER WISE team in navigating the implementation of the intervention at the different stages of a global health crisis. Based on our results, the BETTER WISE approach works best with teams that are characterized by strong networks and good communication, and who value teamwork, including the addition of a PP to provide comprehensive patient-centered care. We suggest considering existing buy-in and communication among team members such as between physicians and potential PPs when implementing BETTER WISE at future sites. These findings are in line with other research that identified the importance of open communication, supportive colleagues, and a collaborative team (Mulvale et al., 2016). For instance, Lukey et al. (2021) confirmed in their systematic review the importance of collaboration in team-based care. Interprofessional care such as between a physician and a PP is a specific example of how to integrate different providers with different professional backgrounds in the provision of coordinated and patient-centered care. Although primary care seems to strive toward interprofessional collaboration and communication, there still seems to be a gap between the ideal and reality (Fox et al., 2021). The BETTER WISE intervention is an example of how interprofessional collaboration can work in the context of prevention and screening.

### Strengths

The strength of this qualitative study is that it integrates various perspectives from primary care providers such as physicians, PPs (who were the “agents of implementation”) and patients (who were the recipients of the BETTER WISE intervention). We used different data collection strategies (focus groups, interviews, and patient feedback forms) that allowed us to triangulate our data and integrate different perspectives. We had a heterogenous sample of primary care clinics that differed in size, localities (including urban, suburban, rural and remote), and mandates (e.g., some were teaching clinics) in different provinces in Canada (Alberta, Ontario, and Newfoundland and Labrador). Another strength is that we were able to capture how the onset of a worldwide health crisis challenged patients’ access to prevention and screening, and how our research team as well as participating clinic teams mitigated these challenges.

### Limitations

Primary care clinics who participated in this study were self-selected and participated voluntarily. Primary care providers interested in implementing a PP could be different from primary care providers who did not volunteer to take part in the BETTER WISE implementation. Furthermore, although we consider that the onset of the COVID-19 pandemic provided an interesting challenge of a “real life” event that could be captured in our study, our results are colored by the context of the implementation taking place during a very specific time in the context of a global health crisis.

## Conclusion

This qualitative study offers insights into barriers and facilitators to the implementation of a prevention and screening program during a global health crisis. Our results suggest that successful implementation was possible despite unforeseen challenges. The main reason for the success was the strong relationships between PPs and their patients, PPs and team members (physicians and managers), and clinic teams led by PPs and the BETTER WISE team. Without the determination of PPs who were the ‘agents of implementation’ and strong interprofessional teamwork, the completion of the intervention would not have been possible. A concise framework such as the CFIR offers a model to other practitioners and researchers who are interested in implementing new approaches in healthcare.

## Data Availability

The datasets generated or analyzed during the current study are not publicly available due to concerns that participants could be identified. Patient data is available from the corresponding author on reasonable request.

## Abbreviations

AB: Alberta, Canada
BETTER: Building on Existing Tools to Improve Chronic Disease Prevention and Screening in Primary Care
BETTER WISE: Building on Existing Tools to Improve Cancer and Chronic Disease Prevention and Screening in Primary Care for Wellness of Cancer Survivors and Patients
CCDPS: Cancer and Chronic Disease Prevention and Screening
CFIR: Consolidated Framework for Implementation Research
COVID-19: Coronavirus Disease
cRCT: Cluster Randomized Controlled Trial
NL: Newfoundland and Labrador, Canada
ON: Ontario, Canada
PP: Prevention Practitioner
WHO: World Health Organization
